# Potential Roles of Fungal Extracellular Vesicles during Infection

**DOI:** 10.1128/mSphere.00099-16

**Published:** 2016-06-29

**Authors:** Luna S. Joffe, Leonardo Nimrichter, Marcio L. Rodrigues, Maurizio Del Poeta

**Affiliations:** a Departamento de Microbiologia Geral, Instituto de Microbiologia Paulo de Góes, Universidade Federal do Rio de Janeiro (UFRJ), Rio de Janeiro, Brazil; b Department of Molecular Genetics and Microbiology, Stony Brook University, Stony Brook, New York, USA; c Centro de Desenvolvimento Tecnológico em Saúde (CDTS) da Fundação Oswaldo Cruz (Fiocruz), Rio de Janeiro, Brazil; d Veterans Administration Medical Center, Northport, New York, USA; University at Buffalo

**Keywords:** Fungal pathogenesis, vesicles, ceramide, fungi, glycolipids, sphingolipids

## Abstract

Extracellular vesicles (EVs) are produced by virtually all cell types. Within the past few years, work in this field has revealed more information about fungal EVs. Fungal EVs have been shown to carry proteins, lipids, pigments, polysaccharides, and RNA; these components are known virulence factors, a fact which supports the hypothesis that fungal EVs concentrate pathogenic determinants. Additionally, recent studies have demonstrated that fungal EVs stimulate the host immune system. In this review, putative roles of fungal EVs are discussed, including their potential as vaccination tools and their possible contribution to pathogenesis in invasive fungal diseases.

## Abstract (precis)

Extracellular vesicles (EVs) are produced by virtually all cell types. Within the past few years, work in this field has revealed more information about fungal EVs. Fungal EVs have been shown to carry proteins, lipids, pigments, polysaccharides, and RNA; these components are known virulence factors, a fact which supports the hypothesis that fungal EVs concentrate pathogenic determinants.

## INTRODUCTION

**FIG 1:**
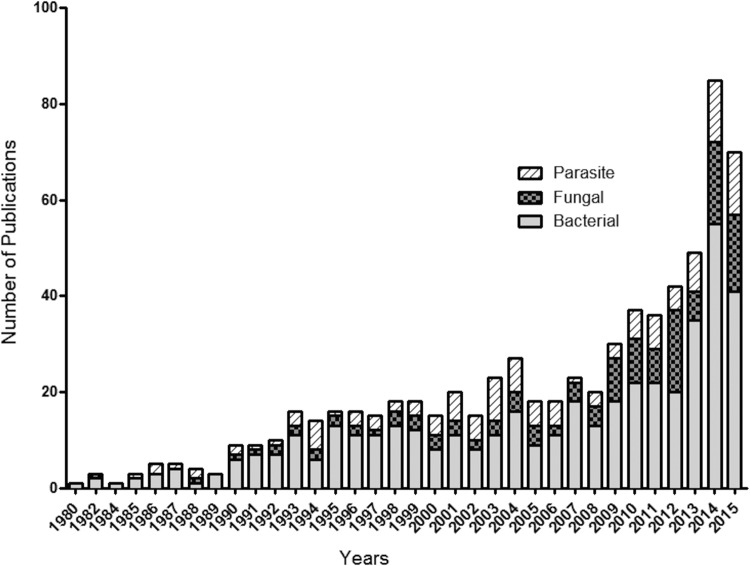
Numbers of publications (peer-reviewed articles, book chapters, reviews, notes, conference reviews) related to EVs produced by bacterial, fungal, and parasite organisms. The Scopus database was used to track microbial EV-related publications from 1980 to 2015. The keywords “fungal/fungi/yeast extracellular vesicles,” “bacterial/bacteria extracellular vesicles,” and “parasite/protozoan/protozoa extracellular vesicles” were used for article searches in titles, abstracts, and keywords.

Extracellular vesicles (EVs) are membranous compartments that are released by all living cells investigated so far. EVs play important roles in nutrition, physiopathogenesis, and cell-to-cell communication (1). EVs are now considered key mediators of immunopathogenesis in bacteria, fungi, and protozoa, which has encouraged research activity in this field (Fig. 1). Currently, the majority of the literature concerning EVs is specific to bacterial cells.

EVs are composed of lipid bilayers, and they range in size from 30 to 1,000 nm (2–5). The dimensions and composition of EVs are determined by their mechanisms of biogenesis, which are classified into compartments: exosomes, microvesicles (also called ectosomes), and apoptotic bodies (6). Exosomes originate from the endocytic pathway and are the smallest vesicles, being approximately 30 to 100 nm in size. Microvesicles are released from cells through membrane shedding, and their dimensions range from 100 to 1,000 nm (6, 7). Lastly, apoptotic bodies are shed from the plasma membrane of cells during programmed death and usually are the largest EVs, ranging from 800 to 1,000 nm in size (8). This review discusses the relationship between the composition and biological functions of fungal EVs and addresses the roles of EVs in fungal pathogenesis and their potential biological activity as therapeutic and prophylactic tools.

## MICROBIAL EXTRACELLULAR VESICLES

**FIG 2:**
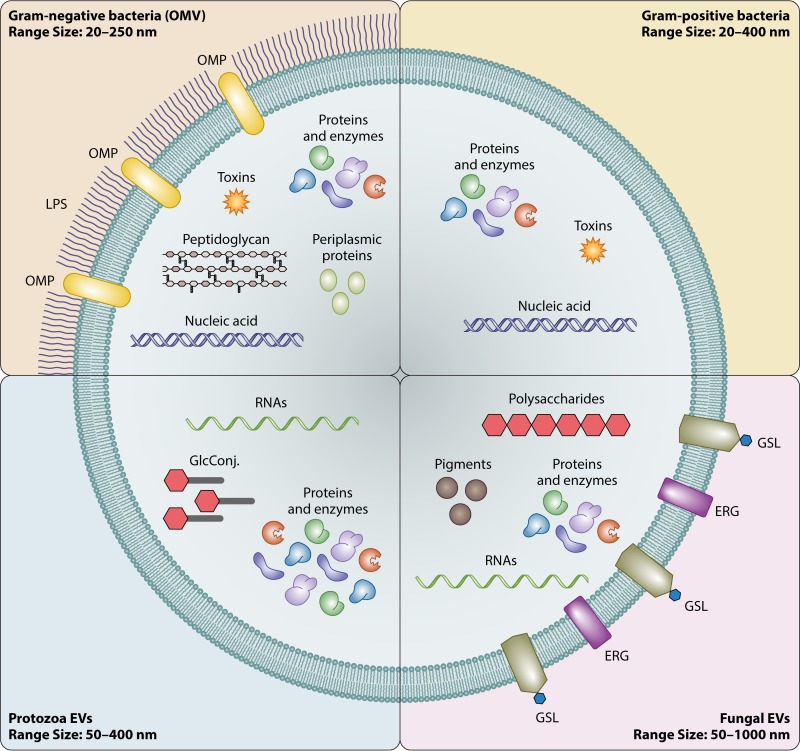
Schematic presentation of EVs produced by different microbial cells. OMP, outer membrane protein; GlcConj., glycoconjugates; GSL, glycosphingolipids; ERG, ergosterol; LPS, lipopolysaccharide.

It is still unclear how EVs are released by cell wall-containing microorganisms, including Gram-positive bacteria, mycobacteria, and fungi (9, 10). EV cargo in these microorganisms includes enzymes, nucleic acids, polysaccharides, pigments, lipoproteins, and toxins (2, 11–14). Both shared EV cargo and organism-specific EV cargo have been characterized, in association with organism-related differences in dimensions (Fig. 2).

## BACTERIAL VESICLES

In bacteria, EVs have been most well studied in Gram-negative cells. The first reports of EV characterization were in *Escherichia coli* in the 1960s (15–18). Gram-negative EVs are also known as outer membrane vesicles (OMVs), due to their site of biogenesis (11, 19). OMVs can increase bacterial resistance to antibiotics and phages by serving as decoy targets for these molecules (19). They can also transfer DNA between cells and carry enzymes that degrade antibiotics (19). Gram-negative OMVs participate in the interaction of bacteria with host cells during infection. They deliver virulence factors, such as toxins, into host cells, including immune cells (19). OMV production is also known to be important for stress responses and nutrient acquisition for bacteria (11). While OMVs have been shown to play a role in bacterial virulence, they can also be harnessed as a potential vaccine tool. Some applications of OMVs as vaccines have already been approved for use in humans or are in clinical trials (20–23).

EVs released by Gram-positive bacteria differ from those released by Gram-negative EVs cells due to the lack of outer membranes and the presence of a thicker peptidoglycan layer (24). The mechanisms by which Gram-positive EVs are released are still not clear, but it is known that they carry cell wall hydrolases and peptidoglycan-degrading enzymes, suggesting that gaps might be formed in cell wall layers in order to release EVs (12, 25–27). Gram-positive species releasing EVs include *Staphylococcus aureus* (28), *Bacillus subtilis* (29), *Streptococcus pneumoniae* (26), *Bacillus anthracis* (12), and *Listeria monocytogenes* (30). Virulence factors of Gram-positive bacteria, including enzymes (β-lactamases, hemolysin, and coagulase) and toxins (12, 28, 31), are also released into the extracellular milieu through EVs. Work done with *B. anthracis* EVs efficiently illustrates how toxin components essential for virulence are released into host cells inside vesicles (12).

EVs released by Gram-positive bacteria are potentially emerging as vaccine components. Rivera and colleagues have shown that toxins released inside *B. anthracis* EVs induced robust immune responses in BALB/c mice that led to higher survival rates in animals challenged with the pathogen (12)*.* Similar results were observed with *Mycobacterium tuberculosis* EVs (32). Mice immunized with mycobacterial EVs induced strong T helper type 1 cell responses (Th1), elicited antibody production, and reduced bacterial burden (32).

## PROTOZOAN VESICLES

Parasite EVs were first described more than 20 years ago, although their relevance for secretory mechanisms has been realized only recently (33). In *Leishmania* spp. and *Trypanosoma cruzi*, EVs play important roles in protein export pathways, macrophage communication, and inflammatory responses (34–36). Proteomic analysis in *T. cruzi* has shown the presence of a great number of proteins containing nucleic acid-binding sites and ribosomal proteins within EVs (37). Different types of small RNAs were also detected in parasite EVs (38). Additionally, there were found to be differences between small RNAs packaged in EVs in each parasite developmental form (38). It has been demonstrated that *T. cruzi* (39) and *Plasmodium falciparum* (40) can transfer genetic information between parasites and from parasites to mammalian host cells. More recently, Fernandez-Calero and colleagues suggested that, under conditions of nutritional stress, a specific type of small RNA is released into EVs that may play a role in parasite-host interaction (41). In *Leishmania* spp., EV release is considered the most important mechanism of protein secretion and mediates delivery of parasite proteins into macrophages to cause production of interleukin-8 (IL-8) (35). Accordingly, EVs may increase parasite virulence; thus, pretreatment of mice with *T. cruzi* EVs followed by intraperitoneal parasite inoculation resulted in mortality indices that were higher than those seen with untreated mice (34). Moreover, EVs induced increased heart parasitism and inflammation through enhanced IL-10 and IL-4 production (34). These data suggested that *T. cruzi* EVs could facilitate parasite dissemination and pathogenic mechanisms (34). More recently, Szempruch and colleagues demonstrated that African trypanosomes release EVs through flagellum-derived nanotube formation, resulting in vesicular fusion with mammalian erythrocytes, membrane disruption, and anemia (42). Together, these results indicate that parasite EVs might participate in mechanisms of either virulence or immune response modulation in parasitic infections.

## FUNGAL VESICLES

EVs produced by fungal cells are peculiar because, like bacterial EVs, fungal EVs must traverse a cell wall in order to be released. The mechanisms of EV release across the complex molecular network of the fungal cell wall are still unknown. Wolf and colleagues have utilized electron microscopy techniques to suggest that EVs interact with cell wall components (43). They showed that single and multiple vesicles simultaneously gain access to the extracellular milieu by crossing of the cell wall (43). The cellular origin of fungal EVs remains unknown. Rodrigues and colleagues suggested that EVs could originate from cytoplasmic subtractions (44). Other studies indicated that multivesicular bodies and membrane budding may also participate in EV formation (43, 45). These mechanisms are compatible with the presence of cytoplasmic proteins lacking secretory signals in EVs (44). The mechanisms of passage through the cell wall are similarly unknown. Proteomic analysis of EVs from *Saccharomyces cerevisiae*, *Histoplasma capsulatum*, *Paracoccidioides brasiliensis*, *Candida albicans*, and *Cryptococcus neoformans* revealed the presence of cell wall-degrading enzymes, suggesting that EV-associated molecules could be present in vesicular membranes and thus exposed, catalyzing cell wall crossing by hydrolysis of structural components (13, 46–49).

EVs transport virulence-associated components to the extracellular medium, suggesting that they are required for fungal pathogenesis (2, 13, 47, 48, 50). In contrast, EVs have been shown to induce protection in experimental models of fungal infection (13). In the following sections, possible roles of fungal EVs as “virulence bags” and their potential as vaccine tools are discussed.

## EV CARGO IN FUNGI

Components of fungal EVs, including lipids (neutral glycolipids, sterols, and phospholipids), polysaccharides (glucuronoxylomannan [GXM] and α-galactosyl epitopes and mannose and N-acetylglucosamine residues), proteins (lipases, proteases, phosphatase, urease, laccase, and many others), and nucleic acids (different RNA species) have been described as virulence factors in different fungal species (2, 13, 14, 47–52). In *C. neoformans* and *C. albicans*, mass spectrometry and high-performance thin-layer chromatography (HPTLC) analysis revealed the presence of glucosylceramide (GlcCer) and sterols in EVs (2, 13). In addition, lipidomic analysis of *P. brasiliensis* EVs revealed the presence of GlcCer, brassica sterol, ergosterol, and lanosterol in different strains (53). GlcCer is a well-known virulence determinant of *C. neoformans*, since mutant cells lacking GlcCer synthase were avirulent in a murine model of cryptococcosis (54). *C. albicans* mutants lacking GlcCer biosynthesis were hypovirulent in BALB/c mice (55). In other fungi, such as *Aspergillus nidulans* and *Fusarium graminearum*, GlcCer is essential for hyphal growth and spore germination (56, 57), highlighting the role of GlcCer in both yeast and filamentous fungi.

Complex carbohydrates, such as GXM, are also exported in fungal EVs (48). In *P. brasiliensis*, EVs contain antigenic α-galactopyranosyl epitopes (50). Peres da Silva and colleagues have shown that residues of mannose and N-acetylglucosamine are present at the surface of *P. brasiliensis* EVs, where they mediate recognition by innate immune receptors (14). These observations might suggest a role for *P. brasiliensis* EVs in pathogen-host communication with the innate immune system, but it remains to be confirmed (14).

Fungal pigments are also found in and exported extracellularly by fungal EVs (48, 52). Melanin granules have dimensions that are comparable to those of EVs (52). In addition, melanin ghosts stained with lipophilic dyes suggested the presence of associated lipids. In fact, purified *C. neoformans* EVs contain electron-dense and dark granules (48) and are able to catalyze melanin synthesis in the presence of l-3,4-dihydroxyphenylalanine (L-DOPA) (52), strongly suggesting that melanin synthesis occurs inside fungal vesicles for further transport to the cell wall (52).

RNA-containing vesicles have been characterized in *C. neoformans*, *C. albicans*, *S. cerevisiae*, and *P. brasiliensis* (51). In these organisms, EVs carry small RNA molecules that are protected by EV membranes against exogenous RNase degradation. Mature tRNAs and mRNAs and noncoding RNAs are also present in fungal EVs (51). Although little is known about the function of RNAs in fungal EVs, it has been suggested that these molecules could participate in cell-to-cell communication, including host cells (51). This is supported by studies in *T. cruzi*, in which tRNA induces changes in host cells, making them more susceptible to infection (58).

Proteomic analysis of fungal EVs revealed the presence of a complex protein composition with multiple functions, including sugar metabolism, cell wall architecture, cell signaling, lipid metabolism, cell growth/division, and virulence (13, 46–49). Although many of these proteins have been shown to be shared in *C. neoformans*, *P. brasiliensis*, *C. albicans*, *H. capsulatum*, and *S. cerevisiae* EVs, species-specific protein molecules have also been abundantly observed (13, 46–49). For instance, enzymes essential to glucuronic acid metabolism were found in *C. neoformans* EVs but not in those of other species (48). EVs produced by fungi contain proteins such as plasma membrane ATPases, cytoskeleton proteins, heat shock proteins (HSP70 and HSP90), adhesins, and antioxidant proteins, including superoxide dismutase and catalase B (2, 13, 47, 48). The activity of urease and phosphatase was also detected in *C. neoformans* EVs (48). Urease is known to be an important virulence factor of *C. neoformans* in enhancing the invasion of host central nervous system (59). Phosphatases, which are surface components in different fungal species (60–62), were detected in other fungal EVs (48, 49).

In the context of all the EV cargo mentioned above, some examples of the involvement of mutants in vesicle formation can help understanding of the importance of fungal EVs. In *C. neoformans*, a Sec6 RNA interference (RNAi) mutant accumulates EVs inside the cells and the levels of some virulence factors, such as those involved in laccase, urease, and polysaccharide secretion, are significantly decreased. As a consequence, its virulence is attenuated (63). This was the first evidence correlating fungal EVs with virulence. Also, in *C. neoformans*, a secretion mutant called *sav1*, lacking a Sec4 GTPase homolog protein of post-Golgi secretion, accumulated EVs inside the cells and showed reduced secretion of proteins (64). In *C. albicans*, phosphatidylserine synthase (*CHO1*) and phosphatidylserine decarboxylase (*PSD1* and *PSD2*) mutants also showed differences in the structures and constitution of EVs (65), but whether this phenotype affects *Candida* virulence is not known. There are some secretion-defective mutants that have been reported in the literature, but the correlation of defects in secretion of EVs with fungal virulence has been studied by our group.

## FUNGAL EVs AS POTENTIAL VACCINES

There are many practical advantages to the general use of EVs as potential vaccine tools. As discussed by Schorey and coworkers (7), EVs offer more-stable conformational conditions for protein components, are able to circulate in body fluids, and show efficient association with antigen-presenting cells (7). However, fungal EVs manifest important particularities. For instance, *C. neoformans* EVs are unstable in the presence of host serum proteins (66).

Studies using EVs as vaccine tools initially included dendritic cell vesicles used for tumor control (67). Recent studies have demonstrated that EV prophylaxis induced increased survival and slower tumor development in mice (68). Development of vaccines using pathogen-derived EVs first included Gram-negative OMVs produced by *Neisseria meningitidis* serogroup B (69–71). Four licensed OMV vaccines are now available (70–72), each of them having been developed for a specific outbreak. Meningococcal group B vaccine (Bexsero), the most recently licensed OMV vaccine, was designed to provide broad protection through combining multiple antigens (72). These EV vaccines were associated with high effectiveness in regions where the circulating and vaccine strains were the same (22, 70, 71, 73). Promising studies focusing on other bacterial species are available in the current literature (20, 21, 74, 75).

Studies on the immunobiological activity of fungal EVs have been performed more recently. In 2011, it was shown that EVs in *Malassezia sympodialis* carry antigens and allergens that modulate the immune system through *in vivo* stimulation of IL-4 and tumor necrosis factor alpha (TNF-α) (76), suggesting an involvement of EVs with atopic eczema (76). Oliveira and colleagues showed that *C. neoformans* EVs are biologically active *in vitro* (77): they stimulate nitric oxide, cytokine (TNF-α, IL-10, and transforming growth factor β [TGF-β]), and fungicidal activity in macrophages (77). This observation might be related to the detection of key immunogens in *C. neoformans* EVs, including heat-shock proteins and GlcCer (78). In fact, human antibodies against *C. neoformans* GlcCer were demonstrated to be fungistatic (45, 78). Similarly, *P. brasiliensis* EVs also stimulated cytokine production in macrophages (14).

Although some reports suggest a protective role of *C. neoformans* EVs, other reports indicate that these compartments can enhance pathogenesis of this fungus. Huang and colleagues have demonstrated that *C. neoformans* EVs are able to cross the brain-blood barrier and accumulate in lesion sites of brain infection (79). In an *in vitro* model of *C. neoformans* infection, EVs facilitated adhesion and transcytosis of *C. neoformans* during interaction with human brain microvascular endothelial cells (79), leading to increased brain infectivity. Finally, pretreatment of mice with *C. neoformans* EVs rendered the mice more susceptible to cryptococcosis (79). Therefore, it is still unclear how *C. neoformans* EVs interfere with either fungal virulence or host stimulation.

There are no fungal vaccines in the clinic, although some animal studies suggest that a fungal vaccine in the clinic may be closer than ever. Wormley and colleagues have suggested the potential protection of using a gamma interferon (IFN-γ)-producing *C. neoformans* strain in a mouse pulmonary infection model (80). Mice were able to resolve the primary infection and showed complete protection against second pulmonary challenge with the *C. neoformans* strain (80). This finding, which was based on utilization of engineered fungi to produce host cytokines in order to induce a protective host immune response, was an important advance in the vaccine field. Another example of a potential fungal vaccine is the *C. neoformans* knockout strain lacking sterylglucosidase 1 (Sgl1), an enzyme responsible for degradation of sterylglucosides. The *Δsgl1* strain was nonpathogenic in an infection model and, interestingly, acted as a vaccine strain, protecting mice when administered prior to challenge with wild-type *C. neoformans* or *C. gattii* (81). This protection may be ascribed to a dramatic accumulation of sterol glucosides (SGs) in the *Δsgl1* mutant (81), and these fungal SGs may act as stronger immunostimulators than plant SGs (82–85). The most interesting observation was that the host was protected against primary and secondary infection even when CD4^+^ T cells were deficient. These data suggest the potential protective effect of an attenuated fungal strain in immunodeficient hosts, such as HIV-positive patients, where cryptococcosis most frequently occurs. Interestingly, if SGs are found in the vesicles of the *Δsgl1* strain, this not only might significantly contribute to the protective effect but also could increase interest in using EVs instead of the live attenuated mutant as a vaccine formulation.

Recently, Vargas and colleagues demonstrated that EVs obtained from *C. albicans* cultures are immunologically active (13). Exposure of macrophages and dendritic cells to purified EVs resulted in nitric oxide production and release of IL-12, TGF-β, and IL-10 in macrophages and of IL-10 and TNF-α in dendritic cells (13). The ability of *C. albicans* EVs to stimulate host cells depended on lipid composition, since EVs from phospholipid synthase mutants differentially stimulated cytokine production in macrophages (65). Treatment of *Galleria mellonella* with EVs before infection with *C. albicans* reduced fungal burden (13). These data indicate the ability of *C. albicans* EVs to modulate the innate immune response and the potential of EVs to interfere with fungal pathogenesis *in vivo* in favor of infection control (13). Together, these results strongly suggest that fungal EVs activate host immunity by multiple mechanisms that may favor the host during fungal infections, reinforcing their potential use as a vaccination strategy.

## CONCLUSION

Fungal diseases are a global public health problem, especially for immunocompromised individuals, such as those affected by HIV infection and cancer (86) (http://www.cdc.gov/fungal/global/index.html). It is estimated that millions of people die every year due to invasive fungal infection (86–88), with a mortality rate comparable to that of malaria (89). The current available antifungal therapies have significant disadvantages: polyene are toxic, azoles induce the development of resistance and are notorious for drug-drug interactions, and echinocandins have a narrow spectrum of activity (86, 90). Thus, there is a need for research and development (R&D) of new antifungals as well as new, alternative preventive strategies to combat fungal diseases.

As discussed above, fungal EVs may represent this new, alternative strategy once we fully understand their cargo and their role as virulence bags. Although there are a variety of examples of the role of fungal EVs in carrying many virulence factors, the relevance of their cargo *in vivo* is incompletely understood. On the other hand, fungal EVs are promising *in vitro* activators of the host immune system against pathogens that operate by inducing cytokine release by innate immune cells, suggesting that they may be a potent tool for vaccine development. Nevertheless, their role in *in vivo* protection is still under study. In bacteria, exciting results that have shown some examples of successful EV vaccines support the idea that fungal EVs could also be used as a protective tool against the most deadly fungal infections. In addition, the concept of therapeutic fungal vaccines is gaining more traction, since there are currently fungal proteins in preclinical phase I studies for vaccine development (91, 92). More studies and more effort are clearly necessary for assessing the role of fungal EVs in the fungus-host interaction and to decipher the mechanisms by which they might stimulate a protective host response.
